# Use of the Environment and Policy Evaluation and Observation as a Self-Report Instrument (EPAO-SR) to measure nutrition and physical activity environments in child care settings: validity and reliability evidence

**DOI:** 10.1186/s12966-015-0287-0

**Published:** 2015-09-26

**Authors:** Dianne S. Ward, Stephanie Mazzucca, Christina McWilliams, Derek Hales

**Affiliations:** Department of Nutrition, Gillings School of Global Public Health, University of North Carolina at Chapel Hill, 2207 McGavran-Greenberg Hall, CB 7461, Chapel Hill, NC 27599-7461 USA; Department of Nutrition, Gillings School of Global Public Health, and Center for Health Promotion and Disease Prevention, University of North Carolina at Chapel Hill, 1700 Martin L. King Jr. Blvd., CB 7426, Chapel Hill, NC 27599-7426 USA; Center for Health Promotion and Disease Prevention, University of North Carolina at Chapel Hill, 1700 Martin L. King Jr. Blvd., CB 7426, Chapel Hill, NC 27599-7426 USA

**Keywords:** Child care, Preschoolers, Nutrition, Physical activity, Measurement

## Abstract

**Background:**

Early care and education (ECE) centers are important settings influencing young children’s diet and physical activity (PA) behaviors. To better understand their impact on diet and PA behaviors as well as to evaluate public health programs aimed at ECE settings, we developed and tested the Environment and Policy Assessment and Observation – Self-Report (EPAO-SR), a self-administered version of the previously validated, researcher-administered EPAO.

**Methods:**

Development of the EPAO-SR instrument included modification of items from the EPAO, community advisory group and expert review, and cognitive interviews with center directors and classroom teachers. Reliability and validity data were collected across 4 days in 3–5 year old classrooms in 50 ECE centers in North Carolina. Center teachers and directors completed relevant portions of the EPAO-SR on multiple days according to a standardized protocol, and trained data collectors completed the EPAO for 4 days in the centers. Reliability and validity statistics calculated included percent agreement, kappa, correlation coefficients, coefficients of variation, deviations, mean differences, and intraclass correlation coefficients (ICC), depending on the response option of the item.

**Results:**

Data demonstrated a range of reliability and validity evidence for the EPAO-SR instrument. Reporting from directors and classroom teachers was consistent and similar to the observational data. Items that produced strongest reliability and validity estimates included beverages served, outside time, and physical activity equipment, while items such as whole grains served and amount of teacher-led PA had lower reliability (observation and self-report) and validity estimates. To overcome lower reliability and validity estimates, some items need administration on multiple days.

**Conclusions:**

This study demonstrated appropriate reliability and validity evidence for use of the EPAO-SR in the field. The self-administered EPAO-SR is an advancement of the measurement of ECE settings and can be used by researchers and practitioners to assess the nutrition and physical activity environments of ECE settings.

## Introduction

Childhood obesity remains a serious public health concern, with about 25 % of children 2–5 years old in the United States (US) classified as overweight or obese (BMI ≥85th percentile) [1]. Excess weight in childhood increases the risk of a child becoming overweight or obese in adulthood [2] and suffering from other chronic health conditions [3–6]. Early childhood has been identified as a critical period for the development of obesity [7–9] and thus, an important target for nutrition and physical activity programs [10].

Child care centers are a particularly important setting to help shape life-long diet and physical activity (PA) behaviors for the prevention of childhood overweight [11]. Recent estimates show that 61 % of preschool aged children attend a center-based child care program, where they spend, on average, 25–30 h per week [12]. According to the American Dietetic Association, children receive 1/2–2/3 of their daily recommended calories while in full time child care [13]. Also, national standards in *Caring for Our Children* recommend that young children receive 90–120 min of moderate- to- vigorous physical activity (MVPA) per 8-hour day in child-care [14]. However, research shows that often children in child care are not meeting nutrition recommendations [15, 16] and that PA levels are low [17]. Experts have called for the development of child care-based interventions to promote better nutrition and PA behaviors and have stressed the importance of comprehensive interventions that target nutrition- and physical activity-related policies and practices [11].

In order to evaluate such interventions, an accurate, comprehensive assessment of the nutrition and PA environments at child care settings is vital. Currently, appropriate measures to assess these environments are limited [18]. One tool that does exist is the Environment and Policy Assessment and Observation (EPAO) instrument [19]. The EPAO was originally created to evaluate the Nutrition and Physical Activity Self-Assessment for Child Care (NAP SACC) intervention, which is based on a set of best practice recommendations. These best practices were developed through an extensive review of all existing national (United States) recommendations and research evidence for healthy nutrition and physical activity environments in center-based child care, and was reviewed by experts in health and child care [20–23]. Although developed for a specific initiative, the EPAO has been utilized widely by researchers because of its comprehensive scope and its link to evidence-based practice [24–32]. The EPAO is an intensive, day-long environmental observation that evaluates all of the provisions and practices occurring within centers and includes a document review of center-based policies. Outcomes from the EPAO provide a comprehensive measure of nutrition and PA environmental characteristics of a child care center, and the instrument has been shown to have strong inter-observer agreement [19].

In addition to the EPAO, two other instruments exist to assess the nutrition and physical activity characteristics at child care centers, but these either evaluate only written policies (WellCCAT), or are limited to physical activity/weather policies (ELEPhANTS). The Wellness Child Care Assessment Tool (WellCCAT) is a 65-item survey completed by center directors about policies (e.g., nutrition education, food/beverage standards, and physical activity) contained in a center’s written documents [33]. The Early Learning Environments for Physical Activity and Nutrition Environments Telephone Survey (ELEPhANTS) is a telephone-administered center director interview to assess aspects of a center’s physical activity practices and includes items about weather and clothing policies, playground size, access, surfaces, shade, topography, and quality; program structure; staff training and behavior; and the sedentary environment [34].

Because the observation format of the EPAO can be costly to implement and requires considerable effort to train and certify data collectors, we modified the EPAO instrument to be completed by center staff using a self-report format (EPAO-SR). To our knowledge, only one self-administered instrument is available to assess both nutrition and physical activity environments [35]. In that survey, directors are asked to report on their center’s nutrition and physical activity practices (23 items), physical environment (3 items, including 11 types of equipment, books and posters), and policies (9 items), as well as the nutrition quality of foods and beverages served (36 items). Evidence used to substantiate validity for most of the items in this instrument was either an interview with the same director who completed the self-report or printed menus; observation by research staff was used to validate only 9 of the survey’s items. We felt that there was a need for a more comprehensive instrument to characterize the nutrition and physical activity environments at child care centers based on strong reliability and validity evidence. Thus, we present the EPAO-SR in this paper, including the instrument’s development, methods for establishing psychometric properties, reliability and validity data, and recommendations for using the new EPAO-SR.

## Methods

### EPAO-SR instrument development

Development of the EPAO-SR instrument occurred in three steps: 1) modification of items from the existing observation-based instrument, 2) review by community advisory group and experts, and 3) cognitive interviews with center directors and classroom teachers.

Development began with a thorough review of the original EPAO by our research group. Items were modified from an observation to a self-report format and questions were added based on the current literature. Additionally, modifications were made to improve item clarity and comprehension based on our extensive field experience with the EPAO and the inter-observer agreement data obtained when the tool was first developed [19]. The SR-based instrument was then distributed to our child care community advisory committee and to two national experts for review. Our advisory committee is comprised of child care professionals, local and state health officials, extension agents, child care providers, and parents, and meets annually to guide NAP SACC and other early care and education activities. The advisory committee and experts were asked to comment on content coverage, and item relevance, format and clarity. Revisions were made based on the feedback from the reviewers.

Finally, cognitive interviews were conducted with a convenience sample of 35 center directors and classroom teachers from across North Carolina. A cognitive interview is a technique commonly used in survey questionnaire development to ensure that the questionnaire designer’s intended interpretation comes across clearly to the respondent and increases the probability that they will respond in a thoughtful manner and give accurate answers [36]. Directors and teachers had participated in a previous study with our research group. Directors and teachers received a subset of the questions by mail prior to the interview and were paid $25 for participating. During the 1-hour telephone interview, trained research staff conducted guided cognitive interviews using a set of questions and prompts developed by our team. Probes from the initial sets of interviews focused on understanding of item content, mental processes used to respond to certain items, and obvious omissions. Later interviews focused more on the layout, structure, and organization of the self-report instrument. Content analysis of the interviews occurred in an iterative manner. When five interviews were completed for a section of questions, issues were summarized and the project team discussed the problems. Revisions were made as needed and questions were reassessed. After three rounds of interviews and revisions the instrument was considered acceptable for further testing.

#### Instrument

The EPAO-SR was designed to evaluate a center’s *provisions* (foods/beverages served, active & sedentary opportunities, PA equipment and the outdoor environment), *practices* (nutrition and physical activity social environment), and *policies* (both nutrition and PA). The EPAO-SR contains close to 800 items. An overview of the items is found in Table 1. Based on experience in the development of the original EPAO instrument and feedback from our advisory committee, we felt that it would be necessary to obtain data from both directors and teachers to obtain the most accurate description of the child care centers’ nutrition and physical activity environments. The self-reporting version (EPAO-SR) is divided into three surveys: the Director Report (completed by the director), the Staff Daily Questionnaire and the Staff General Questionnaire (both staff questionnaires completed by teachers). Each survey contained questions that would be most appropriate for the individual (either director or teacher) to answer. The Director Report asks directors about center-wide nutrition and PA efforts, including parent education and policies for nutrition and PA. The Staff Daily Questionnaire asks classroom teachers to report on daily nutrition and PA provisions and practices on a specific day in a manner similar to a time use diary, while the Staff General Questionnaire asks teachers to report generally on their nutrition and PA practices and infrequent activities such as participation in nutrition and PA training. Most items were presented in a checklist format such that items, or sections, could be skipped if certain activities did not occur or certain types of foods were not served.

**Table 1:** Overview of the EPAO and EPAO-SR and Field Testing Summary

Category	Sample description of items	Questionnaire	Reporter	Day completed			
				1	2	3	4
**Provisions 1**: food and beverages served; active play and sedentary opportunities	• Types of food and beverages served	Staff today survey	Classroom teachers	X	X	X	X
	• Amount of active play time offered, indoor and outdoor	Daily observation tool	Research staff	X	X	X	X
	• Amount of TV, computer, and seated time						
**Provisions 2**: classroom physical environment	• Classroom posters or books featuring food or PA	Staff general survey	Classroom teachers	X		X	
	• Fixed and portable play equipment	Daily observation tool	Research staff	X	X	X	X
	• Amount of space for active play						
**Provisions 3**: center’s physical environment	• Natural features, e.g., trees, open play space	Director survey	Center director	X		X	
	• Presence of vending machines	Daily observation tool	Research staff	X	X	X	X
**Practices**: teacher engagement with children around eating & activity	• Teacher behavior around mealtimes and active play time periods	Staff today survey	Classroom teachers	X		X	
	• Use of food or PA as a reward or punishment	Daily observation tool	Research staff	X	X	X	X
**Policies, Training and Education**: regulations, planned trainings, & formal education	• Policies regarding nutrition or PA	Director survey	Center director	X		X	
	• Nutrition- or PA-related training for center staff	Document review	Research staff				X
	• Parent education around nutrition or PA, e.g., workshops, emails, pamphlets						

### Reliability and validity testing

#### Sample

Child care centers with at least a 2-star rating (North Carolina quality improvement 1–5 star rating scale) were recruited across six counties in piedmont North Carolina via mailed flyers and phone calls from research staff. Eligible centers were identified using the North Carolina Division of Child Development and Early Education website (http://ncchildcare.dhhs.state.nc.us/general/home.asp). Center demographics were obtained during the screening call with the center director. Consent forms were mailed to centers and collected by mail or during the first observation visit (see below.) Directors gave consent for center participation in the study, and parents gave consent for their children to wear accelerometers during the observation period (data not included in this paper). Centers received $100 as an incentive for participation and for completing measures. All methods were reviewed and approved by the University of North Carolina Institutional Review Board.

#### Data collection procedures

A summary of field testing procedures can be found in Table 2. Data were collected across 4 days in each center. Directors identified teachers of classrooms with 3–5 year olds to complete questionnaires and allowed research staff to observe these classrooms for four consecutive days. At least one teacher per center completed the survey, and additional teachers completed the survey if there were multiple teachers per classroom or multiple 3–5 year old classrooms in a center. During the 4-day data collection, classroom teachers completed the Staff Daily Questionnaire for each of the 4 days and the Staff General Questionnaire on two non-consecutive days. Also, directors completed the Director Report on two non-consecutive days. Directors and teachers completed a demographic questionnaire on the first observation day.

**Table 2:** Participant characteristics

Characteristic	Centers (*n* = 50)	
Median star rating^a^	4	
Mean years in operation	16	
CACFP^b^ participation (%)	46	
Mean enrollment (#)	78	
Children receive subsidies (%)	52	
Race/ethnicity of children (%)		
Non-Hispanic White	57	
Non-Hispanic Black	31	
Hispanic	8	
	Directors (*n* = 50)	Teachers (*n* = 124)
Time as center director (years)	10	
Time as classroom teacher (years)		10
Age (years)	44	37
Females (%)	96	100
Highest level of education (%)		
High school or lower	0	10
Some college	25	39
Associates degree	23	22
Bachelor’s degree	40	25
Masters/Doctoral degree	13	3
BMI Category (%)		
Underweight or normal (<25)	35	42
Overweight (25–29.9)	23	22
Obese (≥30)	42	36
Race/ethnicity (%)		
Non-Hispanic White	60	53
Non-Hispanic Black	36	39
Hispanic	2	2

^a^NC Quality Rating System (1–5 stars)

^b^ *CACFP* Child and adult care food program

Research staff trained and certified to conduct the EPAO (observation format) completed direct observations of classrooms with 3–5 year olds for four consecutive days. Observations occurred from the start of the first meal until the majority of the children had left each day. Most observations (*n* = 48) were conducted using one data collector per center. For the remaining two centers, two data collectors conducted the 4-day observations, each observing for 2 days. Documents including policy handbooks, training certificates, menus, and parent education materials were collected from the director for the Document Review. Three data collectors were trained during an intensive half-day workshop that included a review of EPAO items and study protocols, and individuals were certified through an additional half-day observation in a practice center. Percent agreement was calculated between each data collector and the gold standard observer (member of the research team). Staff were certified if agreement was at least 85 % with the gold standard.

### Statistical analysis

Because of the variation in item response format (i.e., continuous or categorical) for the EPAO and EPAO-SR, statistics used to assess reliability and validity evidence at the item level varied. ANOVAs were used to test differences in means across days (e.g., mean of fruit served for days 1, 2, 3, and 4 of observation data), with p-values greater than 0.05 indicating no significant difference of means across days. P-values for these tests are presented in Tables 3, 4, 5, 6 and 7. The following were used to evaluate the evidence where appropriate: percent agreement, kappa, correlation coefficients, coefficients of variation, deviations, mean differences, and intraclass correlation coefficients (ICC). Scale level data were evaluated based on ICCs. Stability of item and scale scores from the new self-report instrument over multiple days was determined by computing reliability statistics for item and scale scores from the self-report across multiple days. Two ICCs are reported, one represents the reliability if all days of available data are averaged to compute an item score (ICC4 or ICC2), the other (ICC1) tells us the reliability if one randomly selected day was used to represent a center’s score for an item. For nutrition and physical activity policies, two-level (no written policy vs. written policy present) percent agreement and Kappa statistics were calculated to compare research staff and center director reports of policies. We used Shrout’s categorizations to evaluate ICC and Kappa statistics: virtually none (0–0.10), slight (0.11–0.40), fair (0.41–0.60), moderate (0.61–0.8), and substantial (0.81–1.0) [37]. Analyses were conducted using SAS v9.2 (Cary, NC).

**Table 3:** Food and beverages served – means, reliability, and validity

				Reliability		Validity			
Food or beverage provision	Source	Means (range day 1–4)	*p*-value means	ICC 1-day	ICC 4-day	Corr (days)	Corr (avg)	Avg dev	Dev as % of mean
Food									
Total grains	Obs	2.75 – 2.96	0.49	0.16	0.42	0.14 – 0.35	0.25	0.20	7.9 %
	Avg staff	2.27 – 2.73	0.02	0.25	0.57				
Meat or alternative	Obs	1.00 – 1.33	0.15	0.14	0.40	0.05 – 0.61	0.32	0.01	1.2 %
	Avg staff	1.01 – 1.09	0.95	0.24	0.56				
Fruit (not juice)	Obs	1.38 – 1.71	0.11	0.28	0.61	0.05 – 0.64	0.53	0.13	9.2 %
	Avg staff	1.20 – 1.48	0.38	0.32	0.65				
Vegetables (non-potatoes)	Obs	0.76 – 0.98	0.13	0.07	0.22	0.29 – 0.63	0.40	0.02	2.7 %
	Avg staff	0.71 – 0.94	0.15	0.06	0.20				
Beverages									
Milk	Obs	1.91 – 2.10	0.62	0.32	0.65	0.60 – 0.69	0.85	0.05	2.4 %
	Avg staff	1.90 – 1.93	0.97	0.45	0.77				
100 % Fruit Juice (oz)	Obs	3.02 – 3.80	0.77	0.38	0.71	0.36 – 0.53	0.51	−0.77	18.0 %
	Avg staff	3.55 – 4.61	0.37	0.36	0.70				
Water (oz)	Obs	3.12 – 3.35	1.00	0.61	0.86	0.48 – 0.58	0.63	−0.60	14.9 %
	Avg staff	3.29 – 4.00	0.78	0.55	0.83				

All provisions are number of offerings, except FJ and water in ounces

*P*-values represent a statistical comparison of the equivalence of means for each day of reporting (means on days 1–4) for each reporter. *P*-values >0.05 indicate no statistical difference between means across days

*ICC* Intraclass correlation, *Corr* Pearson correlation, *Dev* Difference (observation – staff report)

**Table 4:** Physical activity and sedentary opportunities – means, reliability, and validity

				Reliability		Validity			
Physical activity provision	Source	Means (range day 1–4)	*p*-value means	ICC1	ICC4	Corr (avg)	Corr (days)	Avg dev	Dev as % of mean
Outside									
Time outside (min)	Obs	69.4 – 74.5	0.46	0.23	0.54	0.63	0.44 – 0.77	5.8	9.0
	Avg staff	58.7 – 70.4	0.33	0.55	0.83				
Teacher-lead activity (bouts)	Obs	0.8 – 1.1	0.64	0.13	0.37	0.10	−0.03 – 0.29	−1.2	54.5
	Avg staff	2.1 – 2.7	0.44	0.24	0.56				
Inside									
Teacher-led activity (bouts)	Obs	1.1 – 1.4	0.72	0.25	0.58	0.05	0.02 – 0.09	−1.6	57.1
	Avg staff	2.4 – 3.0	0.91	0.24	0.55				
Teacher-led activity (min)	Obs	7.5 – 11.7	0.44	0.11	0.32	0.07	0.00 – 0.13	−22.3	71.7
	Avg staff	23.1 – 42.0	0.58	0.19	0.48				
Music and dance (min)	Obs	4.8 – 7.4	0.60	0.22	0.53	0.32	0.01 – 0.40	−5.7	47.9
	Avg staff	9.5 – 14.3	0.07	0.34	0.67				
Gross motor activity (min)	Obs	2.2 – 5.3	0.45	0.28	0.61	0.23	0.15 – 0.44	−3.3	40.7
	Avg staff	6.4 – 9.9	0.29	0.28	0.61				
TV time (min)	Obs	5.1 – 8.7	0.84	0.39	0.72	0.47	0.24 – 0.71	3.1	84.0
	Avg staff	2.3 – 4.5	0.61	0.41	0.73				
Seated time (min)	Obs	47.9 – 54.3	0.83	0.53	0.82	−0.01	−0.10 – 0.12	13.1	40.3
	Avg staff	25.4 – 38.2	0.01	0.37	0.70				

*P*-values represent a statistical comparison of the equivalence of means for each day of reporting (means on days 1–4) for each reporter. *P*-values >0.05 indicate no statistical difference between means across days. *ICC* Intraclass correlation, *Corr* Pearson correlation, *Dev* Difference (observation – staff report)

**Table 5:** Physical activity equipment and natural environment – means, reliability, and validity

		Means			Reliability		Validity			
PA environment	Source	D1	D2	*p*-value means	ICC1	ICC2	Corr (avg)	Corr (days)	Avg dev	Dev as % of mean
Natural elements	Obs	2.7	2.8	0.99	0.93	0.96	0.70	0.65 – 0.77	−1.50	35.3
	Dir	4.2	4.3	0.83	0.94	0.97				
Fixed equipment	Obs	6.9	7.0	0.73	0.98	0.99	0.51	0.49 – 0.55	−0.28	3.9
	Avg staff	7.1	7.1	0.87	0.73	0.85				
Portable equipment	Obs	6.7	7.3	0.07	0.93	0.96	0.25	0.23 – 0.26	−2.10	23.0
	Avg staff	8.9	9.1	0.61	0.59	0.74				
Sedentary equipment	Obs	1.5	1.8	0.07	0.91	0.95	0.43	0.41 – 0.45	−0.73	31.4
	Avg staff	2.3	2.3	0.88	0.78	0.87				
Inside space for gross motor activity (1–5)	Obs	3.3	3.3	0.83	0.98	0.99	0.03	0.02 – 0.06	0.10	3.1
	Avg staff	3.2	3.3	0.90	0.53	0.69				

*P*-values represent a statistical comparison of the equivalence of means for each day of reporting (means on days 1–4) for each reporter. *P*-values >0.05 indicate no statistical difference between means across days

*ICC* Intraclass correlation, *Corr* Pearson correlation, *Dev* Difference (observation – staff report)

**Table 6:** Nutrition and physical activity social environment – means, reliability, and validity

				Reliability		Validity			
Staff behavior	Source	Means (range day 1–4)	p-value means	ICC1	ICC4	Corr (avg)	Corr (days)	Avg dev	Dev as % of mean
During a meal/snack, the classroom teacher…									
Ate/drank an unhealthy food or beverage	Obs	1.18 –1.70	0.37	0.47	0.78				
	Avg staff	0.90 – 1.09	0.75	0.47	0.78	0.26	0.02 – 0.45	0.58	56.9
Taught children about food eating	Obs	0.60 – 0.76	0.66	0.39	0.72				
	Avg staff	0.46 – 0.86	0.02	0.21	0.51	0.16	0.10 – 0.22	−0.03	4.1
Sat with the children	Obs	1.02 – 1.42	0.33	0.48	0.79				
	Avg staff	1.53 – 1.78	0.47	0.51	0.81	0.28	0.21 – 0.36	−0.36	22.1
Ate the same food as the children	Obs	1.06 – 1.44	0.21	0.44	0.76				
	Avg staff	1.06 – 1.18	0.90	0.60	0.86	0.46	0.38 – 0.53	0.30	26.1
Ate fruit or vegetables in front of children	Obs	0.60 – 0.90	0.24	0.35	0.68				
	Avg staff	0.66 – 0.86	0.47	0.39	0.72	0.39	0.30 – 0.45	0.05	6.4
Encouraged a child to eat more (3 items)	Obs	0.46 – 0.56	0.94	0.23	0.55				
	Avg staff	0.61 – 1.21	0.03	0.33	0.67	0.004	−0.11 – 0.04	−0.49	45.4
During play time, the…									
Teacher joined children active play (3 items)	Obs	1.36 – 1.88	0.42	0.32	0.48				
	Avg staff	0.52 – 0.71	0.10	0.43	0.75	0.42	0.22 – 0.50	−0.05	11.4
Teacher read a book promoting activity	Obs	0.04 – 0.10	0.62	−0.14	−0.33				
	Avg staff	0.17 – 0.21	0.89	0.26	0.58	0.11	−0.01 – 0.39	–	–
Children lost outside time (3 items)	Obs	0.05 – 0.09	0.41	0.29	0.46				
	Avg staff	0.05 – 0.13	0.01	0.16	0.44	0.25	0.07 – 0.35	−0.01	7.3
Children received extra outside time (2 items)	Obs	0 – 0	–	–	–				
	Avg staff	0.02 – 0.05	0.74	0.16	0.43	–	–	–	–
Children lost TV time (2 items)	Obs	0.00 – 0.01	0.51	−0.10	−0.22				
	Avg staff	0.01 – 0.02	0.46	0.35	0.69	0.16	−0.02 – 0.26	−0.01	38.4
Children received extra TV time (2 items)	Obs	0.00 – 0.01	0.35	–	–				
	Avg staff	0.00 – 0.01	0.72	−0.04	−0.17	0.47	0.00 – 0.70	–	–

*P*-values represent a statistical comparison of the equivalence of means for each day of reporting (means on days 1–4) for each reporter. *P*-values >0.05 indicate no statistical difference between means across days

*ICC* intraclass correlation, *Corr* Pearson correlation, *Dev* Difference (observation – staff report)

**Table 7:** Nutrition and physical activity policies – means, reliability and validity

	Reliability					Validity		
	Mean T1 (SD)	Mean T2 (SD)	P_value	ICC1	ICC2	Mean Obs	Corr (T1 – Obs)	*P*_value (T1 – Obs)
Total nutrition policies								
Written	7.49 (6.29)	8.58 (7.70)	0.61	0.76	0.86	6.72 (5.40)	0.27	0.67
General practice	12.20 (6.34)	12.40 (6.92)	0.89	0.70	0.83			
Total physical activity policies								
Written	8.09 (6.24)	8.89 (7.51)	0.58	0.87	0.93	4.57 (5.06)	0.27	0.04
General practice	10.96 (6.01)	11.33 (7.74)	0.80	0.79	0.88			

*P*-values represent a statistical comparison of the equivalence of means for each day of reporting (means on days 1–4) for each reporter. *P*-values >0.05 indicate no statistical difference between means across days

## Results

### Sample characteristics

2A sample of 50 child care centers, 50 center directors, and 124 classroom teachers participated in the study. Characteristics of participating centers, directors, and teachers are described in Table 2. Briefly, 46 % of centers participated in the Child and Adult Care Food Program (CACFP) and 52 % enrolled children who received subsidies towards their enrollment fees. About half (57 %) of children attending the centers were Non-Hispanic White, 31 % were Non-Hispanic Black and 8 % were Hispanic. Center directors were on average 44 years old, and classroom teachers’ average age was 37 years. About half of directors (53 %) had received a bachelor’s degree or higher, while only about a quarter of teachers (28 %) held a bachelor’s degree or higher.

### Provisions: foods and beverages served

Means, reliability and validity estimates for food and beverages served are presented in Table 3. Foods such as grains were served at each meal or snack occasion, while other foods such as fresh fruit, potatoes, and dessert were offered less frequently. Reliability estimates from the teacher report varied, with ICC estimates ranging from 0 to 0.96, and were generally similar to estimates from the observation. Four-day intraclass correlations (ICC_4_) indicate acceptable (fair or better) reliability for most foods after 4 days of reporting. Reliability estimates for potatoes, vegetables, dessert, and whole grains remained low for both observation and teacher report. Correlations between the observation and staff report, ranged from 0.25 to 0.85, with a median of 0.52. Milk, water, and potatoes had the highest correlations (>0.6), while meat and grains had the lowest (<0.33).

### Provisions: active and sedentary opportunities

Results for PA and sedentary opportunities are presented in Table 4. On average, children were provided with about 60 min of outside time daily. Reporting of time outside was most consistent needing only 1 day of reporting to obtain acceptable reliability estimates. Most items had ICC estimates greater than 0.50 using 4 days of reporting. Correlations between the classroom teacher reports and the observation of PA and sedentary opportunities were fair to moderate. Stronger relationships were found for outside time, and TV time. Larger deviations between observations and reporting, as well as lower correlations, were found for teacher-led minutes of PA outside and seated minutes, with teachers over reporting teacher-led minutes of PA outside and underreporting seated minutes.

### Provisions: physical activity equipment and natural environment

Table 5 presents results around physical activity equipment and natural environment features. One-day reliability estimates were high for fixed PA, portable PA, and sedentary equipment, as well as natural environment items. Staff and directors over reported natural, portable, and sedentary equipment by about 30 % compared to the observation. The greatest difference was seen for natural elements, but the correlation between the two measures was high (0.70). For portable PA equipment, the difference between reported and observed was about 20 %, and the correlation between staff and observation was low (0.25), although this small difference (about 2 pieces of equipment) may not be practically meaningful.

### Practices: nutrition and physical activity social environment

Results for the nutrition and physical activity social environment are presented in Table 6. Briefly, unhealthy practices such as teacher consumption of fast food, candy, or donuts were infrequently reported, while healthier practices like teachers eating the same food as the children or sitting with the children during meals were reported on average about once a day.

One- and 4-day reliability estimates ranged from 0.06 to 0.92, with most above 0.3. Validity estimate showed moderate to high agreement between the classroom teacher and observational reports. Higher correlations were seen for the healthier teacher practices, while teacher consumption of fast food and salty snacks had lower correlations between teacher and observation report.

### Policies: nutrition and physical activity

Table 7 presents the means, reliability, and validity estimates for nutrition and physical activity policies. Directors reported whether the policy existed in their center as a formal, written policy or as general practice but not codified into formal policy. More common policies included types of food, beverages, amount of fruit and vegetables served, amount of active time and outdoor play time provided, appropriate clothes and shoes for outdoor play, staff behavior during outdoor play time, and amount/type of TV watched. Less common policies were generally the more in depth policies, such as cooking method for vegetables, promoting staff use of informal nutrition discussion during meals, amount and type of portable play equipment, and nutrition and PA education for parents. High percent agreement and Kappa statistics indicate adequate reliability for most policies, with Kappa statistics ranging from 0.23 to 0.82, and most above 0.5. Validity estimates were higher for policies around amount of fruit and vegetables served, family style dining, food brought by staff, informal nutrition discussion, and amount of time children can spend on computers. Validity estimates were on average higher for nutrition policies than PA policies. Table 7 shows that centers had policies covering about 20 of the 30 nutrition policy topic areas and about 19 of the 26 PA topic areas, with consistent reporting after 1 day of administration and moderate correlation with the observation report.

## Discussion

The EPAO-SR instrument is an important advancement in the measurement of nutrition and physical activity environments in child care settings. To the best of our knowledge, no existing measure of the child care environment is as comprehensive or has undergone as rigorous development and evaluation as the EPAO-SR. The EPAO is based on a comprehensive review of the best practices for nutrition and physical activity environments at child care [19, 20–23]. The EPAO-SR incorporated a careful review of the existing EPAO instrument and included updates based on the current research literature, using cognitive interviews to ensure item clarity. Additionally, evaluation of this new instrument was carefully designed to provide evidence of test-retest reliability and validity of self-report compared to 4 days of direct observation by trained staff. This ensures the quality of the instrument and strengthens the confidence in the findings for assessing the child care environment with the EPAO-SR. The EPAO-SR, in comparison to the other three existing instruments, is a more comprehensive self-assessment tool and with rigorous testing to establish multiple types of reliability and validity evidence.

Data from the current study demonstrated a range of reliability and validity evidence for the individual items contained in the EPAO-SR instrument. Results indicate that reporting from directors and classroom teachers is fairly consistent and similar to the observational data with only one or two administration needed to obtain minimally acceptable reporting of most items. Items that produced strongest reliability and validity estimates included beverages served, outside time, and physical activity equipment. These are factors within the child care environment that are more consistent across days and easier for child care staff to report; for example, physical activity fixed equipment is not likely to change from 1 day to the next and outside time may be a standard amount each day.

Although efforts were made to create the strongest instrument possible, reliability and validity evidence for some items appear low which seems troubling. However, reliability and validity evidence is not contained within a single number and instead is a function both of the item and the type of data collected. Low values (lCC <0.50) certainly help to identify problem areas that may need improvement, but also may identify true lack of variation between people or, in our case, centers. Whole grains served and amount of teacher-led PA had lower reliability and validity estimates. For these items, the reliability estimates were often similar for the child care staff report compared with the observation report. The low reliability or validity estimates may reflect the variable nature of those factors (i.e., day-to-day variability) and/or the inherent difficulties in measuring them, rather than inconsistent or misreporting by child care staff. Additionally, low ICC and correlation estimates can result from low between-subject variation, even with low within-subject variation. In this study, for example, grains were offered at nearly every meal at the majority of centers. This lack of variation likely contributed to the low validity estimate for total grains, since the means across the 4 days were similar between the classroom teachers and between the observation days. Because of these challenges, more days measured or more teachers reporting could improve the estimates for these items.

Differences in reporting also may have occurred due to the subjective nature of some factors, such as teacher behavior during mealtimes and active play. Other items, for example, teachers eating fast food, may have occurred during nap when observers were unable to note this, as observers typically left the center during nap time. Reporting bias by staff may have contributed to the differences noted between certain self-report items and observation data. It is not uncommon for individuals to over report to present a more favorable picture of their behavior. Interesting, we have also seen evidence of underreporting in areas where teachers feel they lack training or low self-efficacy (e.g., support for healthy eating). Although it may be impossible to eliminate bias reporting, survey instructions which stress “no-fault” reporting or “no right answer” may improve accuracy.

Some of the low validity estimates could be strengthened in future administrations by modifying the response format for particular items. For example, in the area of teacher-led bouts of physical activity, correlations between observation and self-report were low, and the mean differences were large. In the EPAO-SR, staff were asked to report the number of teacher-led physical activity bouts in an open response format. This resulted in some higher than expected values. As a modification we have limited the response format so that teachers can select 0–5+ for this item, since more than 90 % of the responses fell within this range. The categorical response seems to be easier for teachers to report and increases the reliability and validity estimates for these items. Thus, changes to instructions and limiting upper boundaries of behavior reporting could improve data quality by reducing burden caused by extreme over-reporting.

Finally, some items, including portable play equipment, may have been stored in areas that were inaccessible to the observer, e.g., stored in another classroom but available for use by multiple classrooms. In this case, the classroom teacher may have reported the presence (availability) of portable play equipment whereas the observer would not have. Continued work may be necessary to address some of these problems and improve upon the instrument.

Although the EPAO–SR was based on an extensive review of authoritative recommendations and research literature, some aspects of the nutrition and physical activity child care environments may need modification based on emerging literature. A few additional areas were noted and will be incorporated into the next version of the EPAO-SR. These areas include more items within staff behavior (enthusiastic role modeling [38–41], verbal praise [41–44], authoritative feeding practices [43–46], and expanded details on the outdoor learning environment, including use of paved, curved pathways and available shade [47, 48], and playground density (child-to-space ratio) [49].

While the sample of child care staff and children in the centers was representative of the racial distribution in North Carolina, the sample was moderately homogenous with 50–60 % of the sample (including directors, classroom teachers, and children) reporting as Non-Hispanic White. Also, the median star rating for the observed centers was high (4 out of 5). While nutrition and physical activity standards are not incorporated in the star rating system, the sample was from centers with higher child care quality standards. The EPAO-SR would benefit from additional testing in more diverse populations, including centers with lower quality ratings, from rural communities, and centers located in lower income neighborhoods. Additionally, there is no observational “gold standard” for some of the measured constructs, including directors’ confidence to make healthy changes within the center and classroom teachers’ valuation of nutrition and physical activity. These are important factors that can influence the child care environment and children’s behaviors, but more work is needed to strength the construct validity evidence for these measures.

The purpose of the EPAO-SR is to evaluate the quality of the child care center’s nutrition and physical activity environments. It uses data gathered from different sources to produce center-level scores. The Director Reportand the Staff General Questionnaire are executed with a single administration. The Staff Daily Questionnaire should be administered by a single teacher on two separate days or by two teachers during a single day. Information from the multiple administrations of the Staff Daily Questionnaire (either more days or more teachers) should be averaged. Finally, the data from the two general questionnaires and the average scores from the daily questionnaire are used to represent the center’s nutrition and PA environments. Because this is a center level assessment, a multi-level model is not appropriate for summarization of these data. Because all children are served the same food and play on the same playground, the overall burden for reporting can be shared by staff and director, and aspects of the center can be evaluated by the most knowledgeable individuals. Because of clustering, different teachers can report food each day decreasing overall burden, while maintaining the quality of the measurement. If there is interest in using the EPAO-SR for classroom level practices and provisions, modifications would be necessary to the protocol to obtain classroom-level estimates instead of center-level.

We encourage use of this instrument by others in the field, either in its entirety or subsections based on individual needs (e.g., only assessing food and beverage provisions.) The use of standardized measures across different studies will strengthen our knowledge of the complex child care nutrition and physical activity environments, as comparisons between studies is easier when similar measures are used. As initially developed, the EPAO was used with a single day administration. Results from this study indicate that, when using the EPAO (observation), multiple days of observation (minimum of two) should be used to improve the validity and stability of the factors within the child care environment. As noted in Table 8, we recommend that the director and at least two teachers be used to obtain the full measure of the nutrition and PA environments within early care and education settings. The Director Report and Staff General Questionnaires can be administered only once, but the Staff Daily Questionnaire requires a minimum of two teachers reporting, or one teacher reporting on 2 days. Optimal practice would be having one teacher report for multiple days (3–4), or multiple teachers reporting for 2–3 days. If fewer days and reporters are employed, the risk will be the loss of information in areas where the occurrence of the provision or behavior is less frequent, difficult to assess, or very similar across ECEs. These decisions will depend upon the needs of the study and researchers.

**Table 8:** Recommendations for future use

Reporter	Content	Days of reporting^a^	
		Minimal	Optimal
Classroom teacher	Provisions *(Nutrition and PA)*		
	a) Foods	2 days	≥4 days
	b) Beverages	1 day	2 days
	c) PA and sedentary time	2 days	≥4 days
	d) Physical environment	1 day	≥2 days
	Practices *(Nutrition and PA)*	1 day	2 days
Center director	Policies, training, and education *(Nutrition and PA)*	1 day	1 day

^a^Multiple days of reporting could be distributed between 2 or more teachers

Since the implementation of this project, we have continued to update and modify the original observation-based EPAO and the EPAO-SR to reflect current NAP SACC best practices and to expand the instrument’s use to other child care settings (e.g., family child care homes). In addition, a scoring system for the EPAO-SR is being developed, similar to that of the initial EPAO observation which will be available along with a copy of the EPAO-SR instrument upon request. We do not necessarily suggest that the EPAO-SR (or even the EPAO in its observational form) is an instrument that should be used for licensing, center-specific funding, or compliance to state or federal regulations. To our knowledge, there are no instruments available which we would recommend for these purposes. Observation is often thought of as a gold standard and may be better for “high-stakes” use in some cases, but we have found that the reliability evidence for observation is similar to teacher and director report in most cases. The EPAO and EPAO-SR are research quality instruments, which can be used to evaluate interventions, test theory, and aggregate center level data to inform policy makers about needed changes or to examine the impact of policy change. We hope that these efforts dedicated to improving the measurement of child care settings will facilitate continued work in this important area.

## Conclusions

Overall, this instrument offers many benefits to the field and can be used in a variety of formats by both researchers and practitioners. A recent meeting of early care and education experts underscored the need for these methods to be “translated and simplified to facilitate use by others,” which is a central goal of the development of this instrument [16]. The EPAO-SR fills this need, as it is both a low-cost and a low-burden tool. The cost of a self-report is much lower than the cost of the traditional gold standard of observation, and dividing the survey amongst directors and classroom teachers reduces the burden of reporting. Additionally, the tool can be used to characterize the child care environment, to understand the relationship between aspects of the child care environment and child weight-related behaviors, and to evaluate interventions targeting the child care environment.
